# Before the Lords' Committee

**Published:** 1890-08-02

**Authors:** 


### Before the Lords' Committee.

(By Our Special Commissioner.)

### IX.—HOSPITAL AND DISTRICT WORK.

On June 24th Mr. Roberts, secretary to the London Hospital, was re-examined on the subject of various items of the accounts. Lord Sandhurst went carefully through every page of the published report, calling attention to every point in the least obscure, or to any item deemed extravagant. Asked what was the Maintenance Fund, Mr. Roberts replied: "It is a special fund started fifteen years ago, and kept up by a special appeal made every five years. We find it far better not to be constantly at the public. Incessant touting does not answer so well as an occasional appeal." Lord Sandhurst: "I quite agree with you." The accounts of the hospital were examined by the Committee, and it was found that they were only occasionally initialled by the Chairman. Lord Cathcart pointed out that in the course of long experience he had always found it customary for the Chairman to initial the books laid before him. Mr. Roberts replied that the auditor went very thoroughly through every item of the accounts, and compared them with the vouchers; the accounts were audited half-yearly, and it took the auditor about six weeks' daily attendance to get through them. Mr. Roberts declined to agree with Lord Kimberley that the accounts could be kept in a more intelligible manner.

Mr. Buxton, Treasurer of the London Hospital, was next examined, and also expressed himself quite satisfied with the system of accounts. The press were freely admitted to the quarterly court, and there were usually two or three reporters present. With regard to the Medical College, it was managed by the College Board and by the Warden. Two visitors were appointed weekly to whom the Warden could refer between the meetings of the Board. They had the power of suspending or dismissing a student, and they sometimes used it. Mr. Buxton is not in favour of a central medical university; but is in favour of the affiliation of provident dispensaries with the hospitals. He believes the difficulty of supporting our great charities is increasing, and that help from government, in the shape of grants for efficiency, will be shortly needed. No new hospitals should be built unless licensed in some way as necessary.

Mr. Nixon, recalled, said the senior medical officers of the hospital had to be members of the Royal College of Surgeons or Physicians, but the house physicians and surgeons need only be graduates in medicine or surgery of a recognised British school. Lord Spencer pointed out that this cut out the Dublin men. Mr. Nixon explained the plan by which the social status of the out-patients is inquired into—a plan, he thought, all hospitals ought to adopt. This was started six years ago, and immediately caused the number of out-patients to drop 75,000 Out of over 22,000 out-patients last year, 822 cases were inquired into; 37 had made false statements, 19 withdrew, and the treatment was terminated in 21 cases. This system of inspection costs about £150 a-year. With regard to the rate of seeing out-patients, Mr. Nixon had drawn up a statement which showed that the physician saw 13 cases an hour, and the clinical assistants about 43 an hour. Some of the Committee, more given to theory than to practice, then tried to make Mr. Nixon acknowledge that there ought always to be plenty of room in the London Hospital. Mr. Nixon said then his management was at fault, for he acknowledged he was quite unable to resist the pressure from without. Lord Kimberley stated it as his opinion that there ought always to be an empty ward waiting in case of a great fire or terrible accident. Your special commissioner could not help wondering what the public would say if an urgent medical case was turned away from the London Hospital and died in the streets, while this ward remained empty waiting for accidents. Some of the noble Lords do not seem in the least to understand the terrible pressure of cases on the London Hospital; and that do what they will, the medical staff and the Committee cannot help occasional overcrowding. Lord Cathcart and Lord Sandhurst seem to be the most practical, and to have personal knowledge of hospital administration.

On Monday last Dr. Stephen Mackenzie was called. He explained the management of the medical college of the London Hospital, and said there were about 460 students on the books. The gross income of the college was between £6,000 and £7,000. Dr. Mackenzie stated that in such a very large town as London he did not think a central medical university could be so useful as the schools in connection with the hospitals. The *esprit de corps* and individuality fostered by separate schools were beneficial. As for the restrictions in qualifications to be held by those seeking staff appointments, he considered that any good man could easily become a member of the Royal College of Physicians or Surgeons, and that, therefore, it was no hardship. Dr. Mackenzie, in answer to Lord Cathcart, stated that he always found the hospital as admirably managed by night as by day, and the wards in the same excellent order; there is neither hurry nor confusion in the wards at night. Dr. Mackenzie was one of the last of the resident medical officers of the London Hospital, and explained that he found that he could not keep in touch with all the cases nominally under his care; he thought the present arrangement, by which each of the visiting staff had his own house physician or surgeon, was far preferable. Nor did he consider it any hardship that the nurses should first see one of the house physicians when ill. With regard to the alleged overcrowding it was true of certain parts of the hospital, but never of the whole; as a whole, the supply of beds fairly met the demand. The system of occasionally putting extra beds in a ward was a lesser evil than borrowing beds from a colleague. The hospital could not well be made larger. Another hospital at Stratford or Bow might reduce the pressure slightly. Though believing firmly in large general hospitals, Dr. Mackenzie refused to acknowledge that special hospitals were an evil; there was room for all, in his opinion. A little conversation on specialism took place between my Lords and Dr. Mackenzie, more amusing than instructive. Dr. Mackenzie desired to put on record his very strong opinion as to the value of the out-patient department. With regard to the nursing of the London Hospital it was admirable, and in his private practice Dr. Mackenzie always employs the nurses, and has never had to complain of them. The point was well summed up thus by the witness: "Nursing is a profession which only women specially qualified can follow. Unless a woman has an interest in and an enthusiasm for the work, she must find it hard; but if she has this enthusiasm it will carry her through all difficulties, and she will even find the work enjoyable."

Mr. Nixon, re-called, read the rule issued to the resident accoucheur with regard to the maternity pupils—"He shall take care no pupil shall attend a midwifery case for the first time unless accompanied by himself, or by a fellow-pupil versed in such cases." Mr. Nixon explained that the maternity department was carried on less as a charity than as a necessity for teaching the students this branch of their work.

Mr. Homersham denied two statements with regard to himself, made by the matron of the London Hospital.

Miss Mansell explained the working of the Metropolitan and National District Nursing Association. All the nurses are ladies; they number about 75, and are selected and appointed by the lady superintendent, and work under her, or her fellow-superintendents. Such patients as can afford it pay a small sum, such as 5s. a-week.

Mr. Lacy explained the working of the East London Nursing Society. The nurses are not ladies. The patients do not pay, but it is under the consideration of the society to appoint a nurse specially for patients able to pay a small sum for her services.

Miss Sprigg contradicted a statement about the New Bond Street Institute, made by Mr. Treves.

